# Dutch utility weights for the EORTC cancer-specific utility instrument: the Dutch EORTC QLU-C10D

**DOI:** 10.1007/s11136-021-02767-8

**Published:** 2021-01-29

**Authors:** Femke Jansen, Irma M. Verdonck-de Leeuw, Eva Gamper, Richard Norman, Bernhard Holzner, Madeleine King, Georg Kemmler, the European Organisation for Research and Treatment of Cancer (EORTC) Quality of Life Group

**Affiliations:** 1 Department of Otolaryngology-Head and Neck Surgery, Amsterdam UMC, Cancer Center Amsterdam, Vrije Universiteit Amsterdam, PO Box 7057, 1007 MB Amsterdam, The Netherlands; 2 Department of Behavioural and Movement Sciences, Section Clinical Psychology, Amsterdam Public Health, van der Boechorststraat 7, 1081 BT Amsterdam, The Netherlands; 3 Department of Psychiatry, Psychotherapy and Psychosomatics, University Hospital Psychiatry II, Medical University of Innsbruck, Innsbruck, Austria; 4 School of Public Health, Curtin University, Perth, Australia; 5 School of Psychology, University of Sydney, Sydney, NSW 2006 Australia; 6 Department of Psychiatry, Psychotherapy and Psychosomatics, University Hospital Psychiatry I, Medical University of Innsbruck, Innsbruck, Austria

**Keywords:** Cancer, Utility, Quality of life, Discrete choice experiment

## Abstract

**Purpose:**

To measure utilities among cancer patients, a cancer-specific utility instrument called the European Organization for Research and Treatment of Cancer (EORTC) QLU-C10D has been developed based on EORTC quality of life core module (QLQ-C30). This study aimed to provide Dutch utility weights for the QLU-C10D.

**Methods:**

A cross-sectional valuation study was performed in 1017 participants representative in age and gender of the Dutch general population. The valuation method was a discrete choice experiment containing 960 choice sets, i.e. pairs of QLU-C10D health states, each health state described in terms of the 10 QLU-C10D domains and the duration of that health state. Each participant considered 16 choice sets, choosing their preferred health state from each pair. Utility scores were derived using generalized estimation equation models. Non-monotonic levels were combined.

**Results:**

Utility decrements were generated for all 10 QLU-C10D domains, with largest decrements for pain (− 0.242), physical functioning (− 0.228), and role functioning (− 0.149). Non-monotonic levels of emotional functioning, pain, fatigue, sleep problems, and appetite loss were combined. No decrement in utility was seen in case of a little or quite a bit impairment in emotional functioning or a little pain. The mean QLU-C10D utility score of the participants was 0.85 (median = 0.91, interquartile range = 0.82 to 0.96).

**Conclusion:**

Dutch utility decrements were generated for the QLU-C10D. These are important for evaluating the cost-utility of new cancer treatments and supportive care interventions. Further insight is warranted into the added value of the QLU-C10D alongside other utility instruments.

**Supplementary Information:**

The online version contains supplementary material available at 10.1007/s11136-021-02767-8.

## Introduction

Current cancer care increasingly asks for economic evaluations to assess whether new cancer treatments or supportive care interventions are cost-effective and should be implemented [1, 2]. This insight is of importance as healthcare costs and other costs related to cancer, such as costs due to absence from work, are high, and choices have to be made regarding the optimal allocation of limited economic resources [2–4]. A commonly used economic evaluation technique is cost-utility analysis, in which the difference in total costs between two or more interventions is compared to the difference in utilities by means of quality-adjusted life years (QALYs) [1].

QALYs are a combination of a persons’ quantity of life and quality of life; one year with an optimal Health-Related Quality of Life (HRQoL) equals to one QALY [1]. The quality component of QALYs can be measured using different techniques, one of which is the use of generic utility measures, such as the EuroQoL-5 Dimensions (EQ-5D) [5, 6] or the Short-Form 6 Dimensions (SF-6D) (based on the SF-36 Health Survey) [7]. These generic utility measures have the benefit that they can be used to measure utilities across disease areas. A disadvantage of the use of such generic utility measures is, however, that HRQoL domains or symptoms specific for a certain disease or complaint are not specifically taken into account. To measure utilities among cancer patients, therefore, a cancer-specific utility instrument has been developed based on the widely used European Organization for Research and Treatment of Cancer quality of life core module (EORTC QLQ-C30) [8] called the EORTC Quality of Life Utility Core 10 Dimensions (EORTC QLU-C10D) [9]. This EORTC QLU-C10D instrument consists of 13 of the 30 items and focuses on 10 of the 15 EORTC QLQ-C30 domains, including four functioning domains (i.e. physical, emotional, social, and role functioning) and six symptom domains (i.e. pain, fatigue, sleep problems, appetite loss, nausea, and bowel problems).

To calculate utilities for the EORTC QLU-C10D, utility weights per individual dimension are needed. Utility weights are commonly based on preferences of the general population [10] and differ among countries and cultures [11]. Currently, utility weights for the EORTC QLU-C10D have been determined for Australia [12], Canada [13], the United Kingdom [14], Germany [15], France [16], Austria, Italy and Poland [17] and valuations for Spain, the United States of America, Singapore and Japan are underway. Although cost-utility analyses are generally required for decision making on reimbursement of new interventions by the Dutch National Health Care Institute [18], Dutch utility weights of the EORTC QLU-C10D have not yet been determined. The aim of this study was therefore to provide Dutch utility weights for the EORTC QLU-C10D using a discrete choice experiment (DCE) in the Dutch general population.

## Methods

### Study design and population

This study was a cross-sectional valuation study in a sample from the Dutch general population. Members from a Dutch online panel who were willing to complete surveys in exchange for a small fee were asked to participate. Quota sampling for age and gender was used in order to get a study population which was representative for the Dutch general population aged 18–80 years. All participants provided consent to participate. According to the Dutch law (i.e. the Medical Research Involving Human Subjects Act (WMO)), this study is not subject to the WMO criteria and, therefore must not undergo review by an accredited medical ethical committee. The Australian valuation study [12], which is similar in study design to this study, was evaluated by a medical ethical committee and approved (University of Sydney Human Research Ethics Committee, 2012/2444). All participants of this Dutch utility study provided written informed consent.

### Valuation survey

Participants were asked to complete an online survey created by the Multi-Attribute Utility in Cancer (MAUCa) Consortium in collaboration with SurveyEngine, a company which specializes in Choice Modelling methods such as DCE. The survey included the DCE, four feedback questions on the perceived difficulty and clarity of the DCE, the participants’ HRQoL (EORTC QLQ-C30) [8], and questions on sociodemographic characteristics (e.g. gender, age, marital status, education level) and clinical characteristics (e.g. chronic diseases). Also, the EQ-5D-5L [19] and the Kessler psychological distress scale [20] were assessed, however, results from these measures were not required for the purpose of this paper.

### EORTC QLU-C10D

The DCE in this study focused on the EORTC QLU-C10D. The EORTC QLU-C10D covers 10 domains using 13 items of the EORTC QLQ-C30 [8], namely physical functioning (item 2 and 3), emotional functioning (item 24), social functioning (item 26 and 27), role functioning (item 6), pain (item 9), fatigue (item 18), sleep problems (item 11), appetite loss (item 13), nausea (item 14), and bowel problems (item 16 and 17), and was developed by the MAUCa Consortium [9]. On each of these domains, participants report a score ranging from level 1 (no impairments in functioning or no symptoms) to level 4 (very much impaired in functioning or very much symptoms).

### Discrete choice experiment

**Fig. 1:**
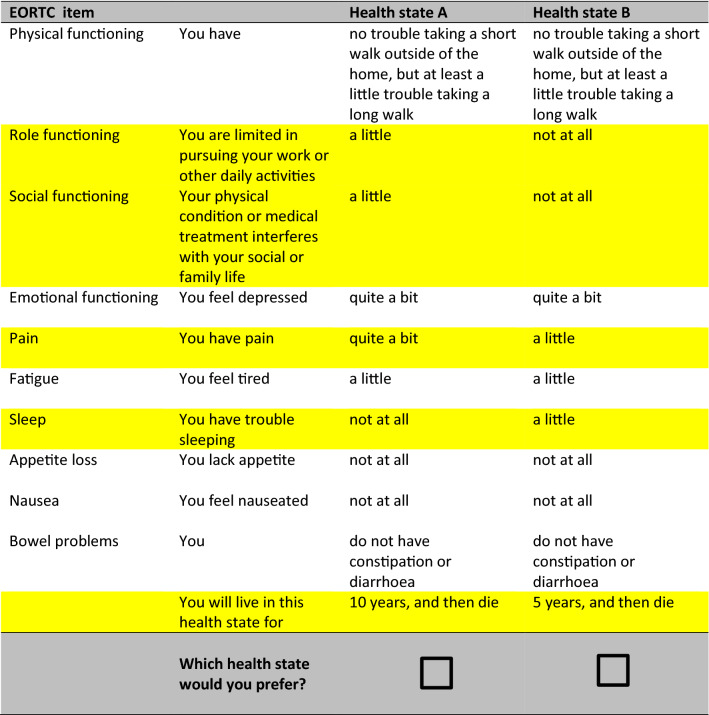
Example of a choice set in the discrete choice experiment

In this DCE, participants considered a series of choice sets, and were asked which of the described health states they would prefer. Based on the choices participants made, utility scores can be derived. In this study, the same DCE methodology was used as described in detail by the Australian valuation study of King et al. [12], and as previously used in the valuation studies in Canada [13], the United Kingdom [14], Germany [15], France [16], Austria, Italy and Poland [17]. The DCE contained 960 choice sets, as formulated in previous research [12], of which each participant randomly received 16 choice sets. Participants were asked in each choice set to choose between two different EORTC QLU-C10D health states, each described by the 10 EORTC QLU-C10D domains as well as the duration of the described health state (i.e. “you will live in this health state for *X* years, and then die”, where *X* = 1, 2, 5 or 10 years). The two health states of a choice set differed on 5 of the 11 (10 domains and one time aspect) attributes, which were highlighted in yellow for convenience of the participant (see Fig. 1 for an example) [21].

### Statistical analysis

Statistical analyses were performed using SPSS version 24 (IBM Corp., Armonk, NY USA) (i.e. for descriptive statistics and Chi-square tests) and STATA version 13 (StataCorp LP, Texas, USA) (i.e. GEE models and mixed logit). Descriptive statistics (frequencies and percentages or mean and standard deviation) were generated for all socio-demographic and clinical characteristics as well as patient-reported outcomes measures and feedback questions. Chi-square tests were used to compare the study population with national statistics [22] for evaluating the representativeness of the study population.

To calculate utility weights, the approach as previously described by Bansback et al. was used [23]. The utility of option j (health status A or B) in choice set s for respondent *i* is described by the following formula:

$$ U_{isj} = \alpha {\text{TIME}}_{isj} + \beta X^{\prime}_{isj} {\text{TIME}}_{isj} + \varepsilon_{isj} , $$

where TIME_*isj*_ is the survival time presented in option *j* (i.e. 1, 2, 5 or 10 years) and *X*ʹ_*isj*_ is a set of dummies related to the levels of the corresponding health state. The errors *ε*_isj_ were assumed to be independent and identically Gumbel distributed. The parameters *α* (scalar) and *β* (vector) were estimated by conditional logistic regression. Regression weights were converted into utility decrements using the ratio of the health state parameters *β* and the time coefficient *α* to reflect the trade-off between HRQoL and length of life, using the same strategy as was applied in the Australian and German valuation studies [12, 15]. To allow for correlated observations within respondents, a random subjects term was included in the model using generalized estimation equation (GEE) models with first-order autoregressive covariance structure. In addition, as some of the utility decrements obtained in the analysis did not show a monotonic pattern, i.e. an increasing EORTC QLU-C10D severity level did not coincide with an increasing decrement in utility weight, in line with previous research [12–17, 24, 25], an additional analysis was performed in which the non-monotonic levels were combined, and the model re-estimated.

Finally, we added a mixed logit model to provide insight into preference heterogeneity in EORTC QLU-C10D domains between respondents. In this model, it was assumed that the coefficients *α* and *β* were drawn from a multivariate normal distribution, thus allowing for heterogeneous preference patterns between respondents. More details may be found in the paper on the Australian valuations [12]. GEE models were, in line with previous valuation studies on the EORTC QLU-C10D [12–17], used to estimate utility decrements. We did not use the mixed logit model to estimate utility decrements, as this model deals with the distribution of parameters rather than with point estimates and consequently its use for estimating utility decrements entails considerable statistical problems (for further information see Gu et al. [26]).

### Sample size calculation

We aimed to include 1000 persons from the general population. The sample size calculation was based on the length of the confidence interval (CI) for the estimated utility decrements. Building on the findings of King et al. (2018) [12] and allowing for the possibility of a slightly larger spread due to a more heterogeneous response pattern (factor 1.2) the half-length d of the 95% CIs for the utility decrements for samples of size *N* = 1000 was estimated to be smaller than 0.05 ([*u* − *d*, *u* + *d*] with *d* ≤ 0.05). This sample size is towards the higher end of the spectrum of so far used sample sizes for DCEs [27].

## Results

**Fig. 2:**
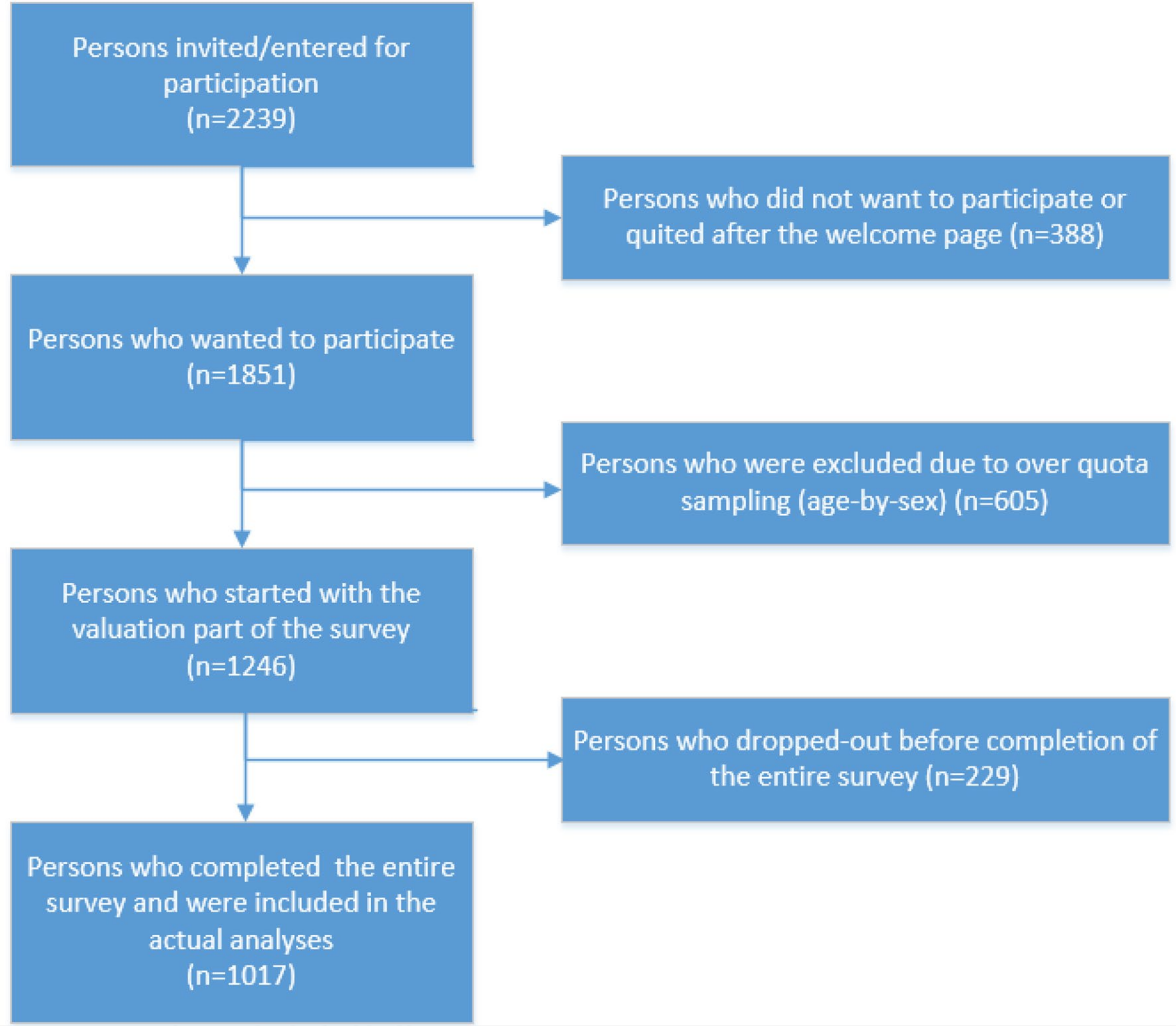
Flow diagram of the study

In total, 2239 members of the Dutch general population were invited to participate in this study, of which 1851 persons wanted to participate. Of these 1851 persons, 1246 persons started the survey and 1017 persons completed all the questions and were consequently included in the analyses (Fig. 2). About half of the participants were male (49.6%). Slightly more than half were married or registered partners (54.5%). Mean age was 48 years and a majority finished secondary or higher education (35.1% and 39.9%, respectively). Almost one third of the participants (*n* = 317, 31.2%) suffered from a chronic disease. Most suffered from arthritis or rheumatoid arthritis (10.5%), followed by asthma, emphysema or chronic bronchitis (10.3%) or diabetes mellitus (7.4%). In total 23 participants (2.3%) had a cancer diagnosis in the past 3 years. This study population was representative for the Dutch general population in terms of gender, age and chronic diseases, but not in terms of education and marital status. Persons who participated were more highly educated and were more often married (Table 1).

**Table 1:** Sociodemographic and clinical characteristics

	General Dutch population	Dutch study population^a^ (*n* = 1017)		Statistics
		*N*	%	
Gender				*χ*^2^ = 0.048; *p* = 0.827
Male	49.9	504	49.6	
Female	50.1	513	50.4	
Age (in years)				*χ*^2^ = 1.360; *p *= 0.93
18–29	19.8	190	18.7	
30–39	15.9	156	15.3	
40–49	17.6	187	18.4	
50–59	19.0	198	19.5	
60–69	15.9	164	16.1	
70–80	11.9	122	12.0	
Education level				*χ*^2^ = 85.330; *p* ≤ 0.001
Elementary	9.0	19	1.9	
Lower	19.9	235	23.1	
Secondary	38.9	357	35.1	
Higher	32.2	406	39.9	
Marital status				*χ*^2^ = 17.601; *p* = 0.001
Not married	37.1	313	30.8	
Married/partnership	49.3	554	54.5	
Divorced/separated	9.8	110	10.8	
Widowed	3.8	40	3.9	
Chronic diseases				*χ*^2^ = 3.382; *p* = 0.066
Yes	33.9	317	31.2	
No	66.1	700	68.8	

^a^Based on national statistics from Statistics Netherlands (CBS)

### Utility decrements

**Fig. 3:**
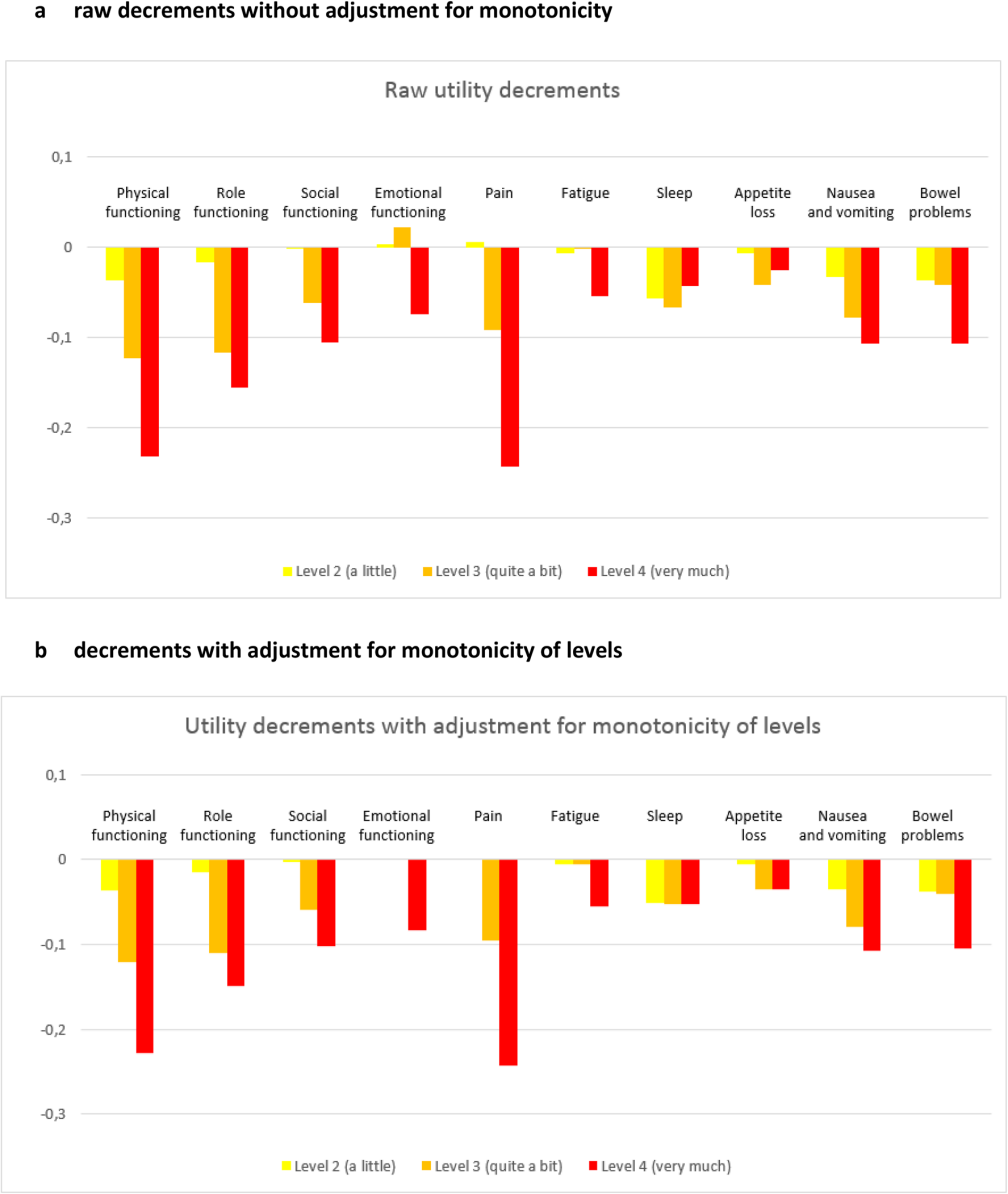
Utility decrements for the Dutch version of the EORTC QLU-C10D. **a** Raw decrements without adjustment for monotonicity. **b** Decrements with adjustment for monotonicity of levels

Findings using GEE models of the raw utility decrements (i.e. decrements of the analysis in which non-monotonicity was allowed) for each level of the 10 EORTC QLU-C10D domains obtained from the DCE are presented in Table 2 and Fig. 3a. The largest utility decrements were shown for pain (level 4 = − 0.243), physical functioning (level 4 = − 0.242), and role functioning (level 4 = − 0.155). Non-monotonicity of levels (i.e. an increasing EORTC QLU-C10D domain level did not coincide with an increasing decrement in utility weight) were observed for the domains on emotional functioning, pain, fatigue, sleep problems, and lack of appetite. Results of the additional analysis in which the non-monotonic levels were combined are shown in Table 2 and Fig. 3b. In this analysis, the largest decrements were also found for pain (level 4 = − 0.242), physical functioning (level 4 = − 0.228), and role functioning (level 4 = -0.149). This was followed by nausea (level 4 = − 0.107), bowel problems (level 4 = − 0.105), social functioning (level 4 = − 0.102), emotional functioning (level 4 = − 0.083), fatigue (level 4 = − 0.055), sleep problems (level 4 = − 0.053) and appetite loss (level 4 = − 0.035). On the emotional functioning domain a decrement in utility was only seen for the highest severity level (i.e. I feel very much depressed), whereas feeling a little or quite a bit depressed were considered comparable as not feeling depressed (i.e. utility decrements of 0). Similarly, no decrement was seen in case of a little pain.

**Table 2:** Utility decrements for Dutch version of the QLU-C10D

Parameter	Severity level	Parameter estimates^a^		Raw utility decrements		Decrements with adjustment for monotonicity of levels	
		*α*	*Β*	Estimate (*β*/*α*)	SE	Estimate	SE
Time		0.461					
Physical functioning (restrictions)	1 (not at all)		REF	0	–	0	–
	2 (a little)		− 0.017	− 0.037	0.024	− 0.036	0.024
	3 (quite a bit)		− 0.057	− 0.123**	0.024	− 0.121**	0.024
	4 (very much)		− 0.107	− 0.232**	0.021	− 0.228**	0.020
Role functioning (restrictions)	1 (not at all)		REF	0	–	0	–
	2 (a little)		− 0.007	− 0.016	0.021	− 0.015	0.021
	3 (quite a bit)		− 0.053	− 0.116**	0.021	− 0.110**	0.020
	4 (very much)		− 0.071	− 0.155**	0.019	− 0.149**	0.018
Social functioning (restrictions)	1 (not at all)		REF	0	–	0	–
	2 (a little)		− 0.001	− 0.002	0.019	− 0.003	0.019
	3 (quite a bit)		− 0.028	− 0.061**	0.019	− 0.059**	0.019
	4 (very much)		− 0.048	− 0.105**	0.017	− 0.102**	0.017
Emotional functioning (restrictions)	1 (not at all)		REF	0	–	0	–
	2 (a little)		0.002	0.003	0.020	0.000	–
	3 (quite a bit)		0.010	0.022	0.022	0.000	–
	4 (very much)		− 0.034	− 0.074**	0.018	− 0.083**	0.015
Pain	1 (not at all)		REF	0	–	0	–
	2 (a little)		0.003	0.006	0.020	0.000	–
	3 (quite a bit)		− 0.042	− 0.092**	0.020	− 0.095**	0.018
	4 (very much)		− 0.112	− 0.243**	0.020	− 0.242**	0.019
Fatigue	1 (not at all)		REF	0	–	0	–
	2 (a little)		− 0.003	− 0.007	0.018	− 0.005	0.016
	3 (quite a bit)		− 0.001	− 0.001	0.020	− 0.005	0.016
	4 (very much)		− 0.025	− 0.054**	0.017	− 0.055**	0.016
Sleep	1 (not at all)		REF	0	–	0	–
	2 (a little)		− 0.026	− 0.056**	0.017	− 0.051**	0.017
	3 (quite a bit)		− 0.030	− 0.066**	0.019	− 0.053**	0.016
	4 (very much)		− 0.020	− 0.043*	0.018	− 0.053**	0.016
Appetite loss	1 (not at all)		REF	0	–	0	–
	2 (a little)		− 0.003	− 0.007	0.019	− 0.005	0.019
	3 (quite a bit)		− 0.019	− 0.041*	0.020	− 0.035*	0.017
	4 (very much)		− 0.011	− 0.025	0.018	− 0.035*	0.017
Nausea	1 (not at all)		REF	0	–	0	–
	2 (a little)		− 0.015	− 0.033	0.018	− 0.035	0.018
	3 (quite a bit)		− 0.036	− 0.078**	0.019	− 0.079**	0.019
	4 (very much)		− 0.049	− 0.106**	0.017	− 0.107**	0.017
Bowel problems	1 (not at all)		REF	0	–	0	–
	2 (a little)		− 0.017	− 0.037	0.019	− 0.038*	0.018
	3 (quite a bit)		− 0.019	− 0.041*	0.020	− 0.041*	0.020
	4 (very much)		− 0.049	− 0.106**	0.017	− 0.105**	0.017
QIC				18,535.896		18,527.433	
QICC				18,521.158		18,513.676	

*QIC* Quasi Likelihood under Independence Model Criterion, *QICC* Corrected Quasi Likelihood under Independence Model Criterion

^a^Parameter estimates obtained from GEE models; *α* denotes the time parameter and *β* the health state parameters

*p < 0.05; **p < 0.01

Findings of the mixed logit analysis are shown in Online Appendix A. Regarding non-monotonicity, a similar pattern was found as for the GEE models. The majority of the estimated standard deviations of the model parameters were significantly greater than zero (27 of 31 parameters), reflecting considerable heterogeneity in individual respondents’ preferences.

### EORTC QLU-C10D utilities of the Dutch general population

Based on the utility decrements associated with the levels of QLU-C10D for an individual patient (as self-reported by that patient in their QLQ-C30 responses), the utility value of an individual person can be obtained by subtracting the corresponding utility decrements for each EORTC QLU-C10D domain from 1. Utility decrements derived from the GEE models with adjustment for monotonicity of levels were used. The SPSS syntax code for this calculation is provided in Online Appendix B. In case a patient is in optimal HRQoL, i.e. the patients scores level 1 (no impairments in functioning or no symptoms) on each of the EORTC QLU-C10D domains, the total utility score equals 1. A person who scores level 4 (very much impairment or very much symptoms) on all EORTC QLU-C10D domains will obtain a score of − 0.159. This is considered to be worse than death, as death equals a score of 0. The person with heath state A in Fig. 1 has a utility score of 0.88 (i.e. 1 minus 0.015 (a little limitation in role functioning) minus 0.003 (a little limitation in social functioning) minus 0 (quite a bit limitation in emotional functioning) minus 0.095 (quite a bit pain) and minus 0.005 (a little fatigue)). The mean utility score of this study population, which was representative for the Dutch general population in terms of age and gender, was 0.85 (median = 0.91) The interquartile range ranged from 0.82 to 0.96, indicating than half of all participants had a utility score between 0.82 and 0.96.Two participants (0.2%) had the lowest possible score (i.e. − 0.159) and 152 (14.9%) had the highest possible score (i.e. 1).

### Feedback on the discrete choice experiment

Completing the survey took on average 15.6 min of the participant’s time (median = 13.2 min; interquartile range 9.5–18.7 min). To provide insight into the experienced difficulty with the DCE, four evaluation questions were assessed. Of all participants, 43% reported difficulty choosing between the two different health states. However, only 6% of the participants thought the presentation of the health states was unclear, whereas 73% reported that this was clear. Participants used different strategies to choose between the different health states, e.g. focusing on the highlighted aspects (i.e. the 5 aspects that differed between the two health states) (27%), thinking about most of the aspects (22%) or focusing on a few aspects (21%).

## Discussion

This study aimed to investigate Dutch utility decrements for the EORTC QLU-C10D using a DCE among the Dutch general population. Information on Dutch utility decrements for the EORTC QLU-C10D is highly relevant, as in the Netherlands, cost-utility analyses alongside clinical trials are frequently performed. The conduct of cost-utility analyses is increasingly seen as mandatory for decision-making on reimbursement of new interventions in the Netherlands [18]. In this study, utility decrements were generated for all of the 10 EORTC QLU-C10D domains, with highest decrements for pain, physical functioning and role functioning, followed by nausea, bowel problems, social functioning, emotional functioning, fatigue, sleep problems and appetite loss.

Findings on Dutch decrements for the EORTC QLU-C10D are slightly different from the utility decrements found in the Australian, Austrian, Canadian, French, German, Italian, Polish and United Kingdom general population [12–17]. In the Dutch population, the largest decrement was observed for pain followed by physical functioning and role functioning, whereas in all other valuation studies, except for the Polish valuation study [17], the largest decrement was seen for physical functioning followed by pain. A reason why impairments in physical functioning may have less impact on the utility outcome in the Netherlands might be that the Netherlands is, compared to the other countries, more densely populated, has less rural areas and has a cycling culture. Consequently, patients who experience impairment in walking (the physical functioning domain of the EORTC QLU-C1D is operationalized as walking) may still be able to cycle and therefore impairments on the physical functioning domain may have less impact on a person’s daily life. However, so far, no studies have been performed to test this hypothesis.

The fourth largest decrement in this Dutch valuation was found for the cancer-specific Nausea domain. This is in line with findings of the Austrian, German and Polish valuation study [15, 17], but in contrast to the Australian, Canadian, Italian and United Kingdom valuation study which reported that emotional functioning was the fourth most impactful domain [12–14, 17]. This is remarkable as utility decrements for the emotional functioning domain are in both the Dutch and German general population quite low. In the Dutch population utility decrements were even only seen in case a person felt very much depressed, while no decrements were found for being a little or quite a bit depressed. It was previously hypothesized in the German valuation study [15] that the difference in utility decrements may be due to the used wording. In the German version the EORTC item on “feeling depressed” was translated into wording that resembles the English terminology for “feeling cast down” or “moody”. This same discrepancy in wording is also seen in the Dutch EORTC translation, which uses the term “neerslachtig” instead of “depressief voelen”. In the Dutch translation of the EQ-5D, however, also less severe terminology was used for the domain on anxiety/depression (i.e. “angstig of somber” comparable to the English wording “anxious or gloomy”) [28]. Despite using this less severe terminology, the Dutch valuation study of the EQ-5D did show large utility decrements for the anxiety/depression domain, higher than the EQ-5D domains on mobility, self-care and usual activities. The Dutch utility decrement of the EQ-5D domain on depression/anxiety was even larger than those found in the United Kingdom population (i.e. − 0.325 and − 0.236 in case of feeling extremely anxious or depressed) [28]. It thus remains unclear why relatively low utility decrements were found for the domain on emotional functioning in the Dutch EORTC QLU-C10D valuation study. More generally, it also suggests that methodological artifact may contribute to between-country differences in utility decrements via that vagaries of translation.

The ranking of the other six EORTC QLU-C10D domains in this Dutch valuation study also slightly differs from the previous valuation studies. These differences support the use of country-specific utility weights rather than overall (not country-specific) utility weights in decision making in a certain country. In the Netherlands, the EQ-5D with 5 levels is recommended in the Dutch National Health Care Institute (ZIN) guideline as the utility measure of first choice since 2015 [18]. They, however, acknowledge that, disease-specific utility instruments, such as the EORTC QLU-C10D, provide additional insight into the effects of new interventions on HRQoL domains and symptoms specific for a certain disease. Further insight into the sensitivity of the EORTC QLU-C10D and the added value of the EORTC QLU-C10D alongside the EQ-5D is, however, needed.

This study was the first that provided Dutch utility weights for the EORTC QLU-C10D. A previous study by Versteegh et al. [29] did already report on Dutch utility weights for an instrument based on 8 of the 15 EORTC QLQ-C30 domains, the QLQ-C30-PBM. However, the valuation study of the QLQ-C30-PBM used a different set of key items from the EORTC QLQ-C30 than the EORTC QLU-C10D, hampering direct comparability of utilities derived from the two utility instruments. Six EORTC QLQ-C30 key items were comparable between the EORTC QLU-C10D and the QLQ-C30-PBM, namely item 2, 6, 9, 14, 18 and 27. To measure emotional functioning, however, Versteegh et al. [29] used the item of worry (item 22), whereas in the EORTC QLU-C10D, the item on depression is used (item 24). In addition, Versteegh et al. [29] included an item on cognitive functioning (item 20), whereas in the present study additional items were included on sleep problems, appetite loss and bowel problems. In adddition, the EORTC QLU-C10D uses two items to measure each of the physical-functioning and social-functioning domains, whereas Versteegh et al. [29] included only one for each of these domains. Another Dutch study [30] investigated the performance of algorithms for mapping EORTC QLQ-C30 scores onto the EQ-5D, which can be used to calculate EQ-5D utility scores in case EQ-5D data is not collected. This objective differs from the objective of our study on Dutch utility weights for the EORTC QLU-C10D in which we aim to provide a cancer-specific utility instrument which can be used alongside the EQ-5D.

A strength of this study is that we included 1017 participants representative in age and gender to the Dutch general population. In addition, standardized valuation methodology was used [12–15], which enables sound comparisons across countries. However, the study population was not representative for the Dutch general population in terms of education level and marital status. Persons who participated in this study were more highly educated and more likely to be married. Another limitation is that non-monotonicities were encountered (i.e. an increasing EORTC QLU-C10D domain level did not coincide with an increasing decrement in utility weight) on emotional functioning, pain, fatigue, sleep and loss of appetite. However, none of these non-monotonicities reached statistical significance. Imposed constraints were used to remove non-monotonicities in the final analyses, as has been done in all previous QLU-C10D valuations [12–17] and for other utility algorithms [24, 25].

In conclusion, utility decrements could be generated for all of the 10 EORTC QLU-C10D domains, with highest decrements for pain, physical functioning, role functioning, nausea and bowel problems.

These utility decrements are, alongside usage of other generic instruments as the EQ-5D-5L, important for studies that aim to evaluate the cost-utility of cancer treatment or supportive care interventions in cancer care, as cancer-specific HRQoL domains and symptoms are taken into account. Utility weights for the EORTC QLU-C10D using the MAUCa standardized valuation methodology have so far been reported among Australian, Canadian, German, French, Austrian, Italian, Polish and United Kingdom populations, with multiple other countries underway, enabling international comparison of findings. Further insight is warranted into the clinical validity of the EORTC QLU-C10D, which will show whether it provides any advantage over commonly applied generic utility instruments such as the EQ-5D.

## Supplementary Information

Below is the link to the electronic supplementary material.

Electronic supplementary material 1 (DOCX 16 kb)

## Data Availability

The data will not be published in an open repository. However, data is available upon reasonable request.

## Code availability

Code is available upon reasonable request.
