# Supplementary material for: Dutch utility weights for the EORTC cancer-specific utility instrument: the Dutch EORTC QLU-C10D
Source: Qual Life Res. 2021 Jan 29;30(7):2009–19. doi: 10.1007/s11136-021-02767-8 (PMC8233279; doi:10.1007/s11136-021-02767-8)
Supplement: Supplementary file 1 — Electronic supplementary material 1 (DOCX 16 kb) [file 11136_2021_2767_MOESM1_ESM.docx]

**Appendix A. Mixed logit**

| Dimension | Level | Coefficient (SE) | Standard deviation (SE) |
| --- | --- | --- | --- |
| Duration | Linear | 1.123 (0.050)*** | 0.630 (0.026)*** |
| Physical functioning x duration | 2 | -0.082 (0.019)*** | 0.121 (0.033)*** |
|  | 3 | -0.117 (0.020)*** | 0.123 (0.025)*** |
|  | 4 | -0.171 (0.018)*** | 0.142 (0.025)*** |
| Role functioning x duration | 2 | -0.036 (0.018)** | 0.160 (0.024)*** |
|  | 3 | -0.126 (0.019)*** | 0.069 (0.030)** |
|  | 4 | -0.132 (0.016)*** | 0.046 (0.031) |
| Social functioning x duration | 2 | 0.014 (0.018) | 0.168 (0.028)*** |
|  | 3 | -0.053 (0.018)*** | 0.095 (0.037)** |
|  | 4 | -0.132 (0.017)*** | 0.142 (0.026)*** |
| Emotional functioning x duration | 2 | 0.017 (0.016) | 0.110 (0.026)*** |
|  | 3 | 0.014 (0.018) | 0.160 (0.024)*** |
|  | 4 | -0.112 (0.018)*** | 0.196 (0.026)*** |
| Pain x duration | 2 | 0.032 (0.018)* | 0.173 (0.025)*** |
|  | 3 | -0.044 (0.019)** | 0.193 (0.027)*** |
|  | 4 | -0.228 (0.019)*** | 0.227 (0.022)*** |
| Fatigue x duration | 2 | -0.008 (0.017) | 0.114 (0.026)*** |
|  | 3 | -0.016 (0.018) | 0.083 (0.035)** |
|  | 4 | -0.080 (0.016)*** | 0.071 (0.032)** |
| Sleep x duration | 2 | -0.020 (0.017) | 0.110 (0.029)*** |
|  | 3 | -0.041 (0.018)** | 0.039 (0.031) |
|  | 4 | -0.032 (0.018)* | 0.167 (0.029)*** |
| Appetite loss x duration | 2 | 0.000 (0.018) | 0.157 (0.037)*** |
|  | 3 | -0.048 (0.018)*** | 0.070 (0.028)** |
|  | 4 | -0.058 (0.016)*** | 0.049 (0.034) |
| Nausea x duration | 2 | -0.025 (0.017) | 0.089 (0.039)** |
|  | 3 | -0.091 (0.018)*** | 0.074 (0.029)** |
|  | 4 | -0.114 (0.018)*** | 0.200 (0.022)*** |
| Bowel problems x duration | 2 | -0.013 (0.017) | 0.176 (0.023)*** |
|  | 3 | -0.050 (0.018)*** | 0.024 (0.050) |
|  | 4 | -0.126 (0.017)*** | 0.126 (0.026)*** |
| Log likelihood | -7844 |  |  |
| AIC | 15812 |  |  |
| BIC | 16332 |  |  |

Abbreviations: AIC, Akaike Information Criterion; BIC, Bayesian Information Criterion

* p<0.05 ** p <0.01 *** p <0.001

**Appendix B. SPSS code to calculate EORTC QLU-C10D scores, based on the Dutch utility weights**

* Dutch EORTC QLU-C10D value sets, Quality of Life Research.

* Femke Jansen, Irma M. Verdonck-de Leeuw, Eva Gamper, Richard Norman, Bernhard Holzner,

* Madeleine King, Georg Kemmler.

* on behalf of the European Organisation for Research and Treatment of Cancer (EORTC) Quality of Life * Group.

* Corresponding author: Femke Jansen, Department of Otolaryngology-Head and Neck Surgery,

* Amsterdam UMC, Vrije Universiteit Amsterdam, Cancer Center Amsterdam, Amsterdam, The

* Netherlands.

* f.jansen1@amsterdamumc.nl .

**************************************************************************************************************************************************************************

* Example code for converting EORTC QLQ-C30 data into QLU-C10D utility scores.

* Written for SPSS by Femke Jansen, July 2020, based on SPSS syntax of Gamper EM et al. Qual Life Res

* 2020.

* More information on the EORTC QLU-C10D can be found in the following papers:

* King MT et al. Qual Life Res 2016.

* Norman R, et al. Qual Life Res 2016.

* The utility algorithms reported in this code are based on the monotonitcity adjusted values as

* reported by Jansen et al. in:

* Dutch utility weights for the EORTC cancer-specific utility instrument: the Dutch EORTC QLU-C10D.

* This code is written for SPSS users.

**************************************************************************************************************************************************************************

* Assumption: For this codes to work, it is assumed that the EORTC QLQ-C30 code is:

* set up as thirty columns, labelled qlq1-qlq30 (in the order as given in the questionnaire),

* each of which can take one of four values 1-4, where

* 1 = "Not at all".

* 2 = "A little".

* 3 = "Quite a bit".

* 4 = "Very much".

*************************************************************************************

*************************************************************************************

* Dutch EORTC QLU-C10D scoring algorithm.

IF (qlq2=1) pf = 0 .

IF (qlq2>1) pf = 0.036 .

IF (qlq3>1) pf = 0.121 .

IF (qlq3>2) pf = 0.228 .

EXECUTE.

IF (qlq6=1) rf = 0 .

IF (qlq6=2) rf = 0.015 .

IF (qlq6=3) rf = 0.110 .

IF (qlq6=4) rf = 0.149 .

EXECUTE.

IF (qlq26=1 & qlq27=1) sf = 0 .

IF (qlq26=2 | qlq27=2) sf = 0.003 .

IF (qlq26=3 | qlq27=3) sf = 0.059 .

IF (qlq26=4 | qlq27=4) sf = 0.102 .

EXECUTE.

IF (qlq24=1) ef = 0 .

IF (qlq24=2) ef = 0 .

IF (qlq24=3) ef = 0. .

IF (qlq24=4) ef = 0.083 .

EXECUTE.

IF (qlq9=1) pa = 0 .

IF (qlq9=2) pa = 0 .

IF (qlq9=3) pa = 0.095 .

IF (qlq9=4) pa = 0.242 .

EXECUTE.

IF (qlq18=1) fa = 0 .

IF (qlq18=2) fa = 0.005 .

IF (qlq18=3) fa = 0.005 .

IF (qlq18=4) fa = 0.055 .

EXECUTE.

IF (qlq11=1) sl = 0 .

IF (qlq11=2) sl = 0.051 .

IF (qlq11=3) sl = 0.053 .

IF (qlq11=4) sl = 0.053 .

EXECUTE.

IF (qlq13=1) ap = 0 .

IF (qlq13=2) ap = 0.005 .

IF (qlq13=3) ap = 0.035 .

IF (qlq13=4) ap = 0.035 .

EXECUTE.

IF (qlq14=1) na = 0 .

IF (qlq14=2) na = 0.035 .

IF (qlq14=3) na = 0.079 .

IF (qlq14=4) na = 0.107 .

EXECUTE.

IF (qlq16=1 & qlq17=1) bo = 0 .

IF (qlq16=2 | qlq17=2) bo = 0.038 .

IF (qlq16=3 | qlq17=3) bo = 0.041 .

IF (qlq16=4 | qlq17=4) bo = 0.105 .

EXECUTE.

COMPUTE QLUC10D_Dutch = 1- (pf + rf + sf + ef + pa + fa + sl + ap + na + bo) .

FORMATS QLUC10D_Dutch (F8.3).

EXECUTE.

*************************************************************************************

*************************************************************************************

* The new variable QLUC10D_Dutch is a utility score where full health (i.e. level 1 in each of the utility levels)

is scored at 1, and the minimum score (i.e. each utility level is at 4) is -0.159.

*This score is considered worse than death as death equals a score of 0.

*************************************************************************************
